# Supplementary material for: SARS-CoV-2 viral protein Nsp2 stimulates translation under normal and hypoxic conditions
Source: Virol J. 2023 Mar 30;20:55. doi: 10.1186/s12985-023-02021-2 (PMC10060939; doi:10.1186/s12985-023-02021-2)

Supplemental Information

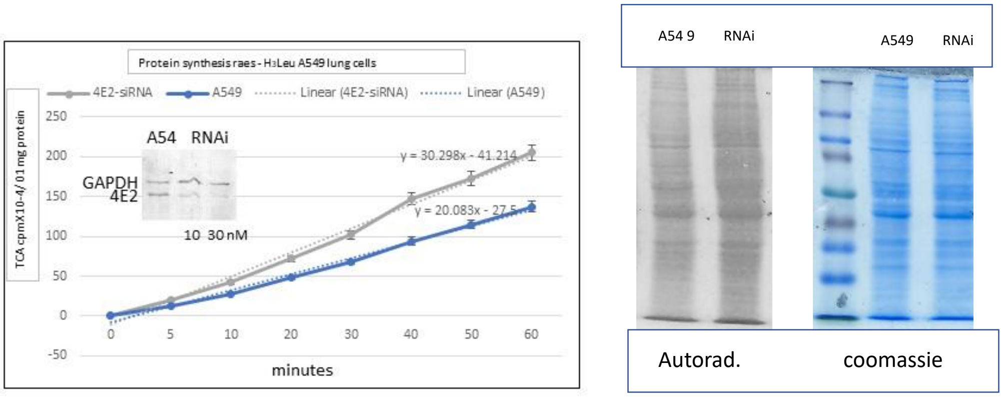

Supplemental Figure 1. Measurement of Protein Synthesis rates in A549 cells depleted of eIF4E2

A549 cells have been transfected with siRNA (SENSE: CGAGACAAGAAUCAGAGCAtt, Ambion/Life Technologies) for 24h. The inset shows the effective dose-depletion of eIF4E2. 10^5 A549 cells with or without 4E2-siRNA were each plated in 12 wells of a 24-well plate with 1ml complete D-MEM and 10% FCS (duplicate samples). The next day the medium was replaced with medium containing 5µCi/ml L-[3,4,5-3H(N)]-Leucine (150 Ci/mmol - NEN), and sequential aliquots were removed at indicated intervals. After solubilization with 0.5 ml 1%SDS, 10% TCA insoluble material (0.1 mg Protein) was collected on 2.5 cm GFA filter. Following washing with 90% EtOH, the filters were air-dried and placed in scintillation vials for counting with OptiScint LLT NPE-Free Scintillation cocktail in a Beckman LS6500 counter. Note that the difference in PS rates is highly significant ($P<10^{-6}$). 10 µg of protein isolated at 1h was processed by 8%PAGE/SDS for fluorographic auradiography with PPO.

Supplemental Figure 2. Nsp2-cells demonstrate higher cap- and HCV-dependent translation under normal and hypoxic conditions. Expression of Renilla and Firefly luciferases from capped-Renilla-HCV-IRES-FF Luciferase mRNA in control and Nsp2 cells grown under normal (white bars) or hypoxic (grey bars) conditions. Graphs represents mean of Renilla units (expressed from the capped mRNA) **(left panel),** and Firefly (expressed from the HCV IRES mRNA) **(right panel)** luciferase signals (±SD, n=3).

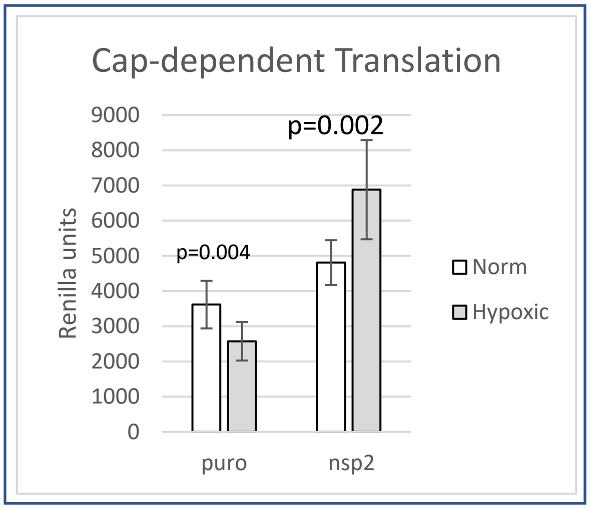

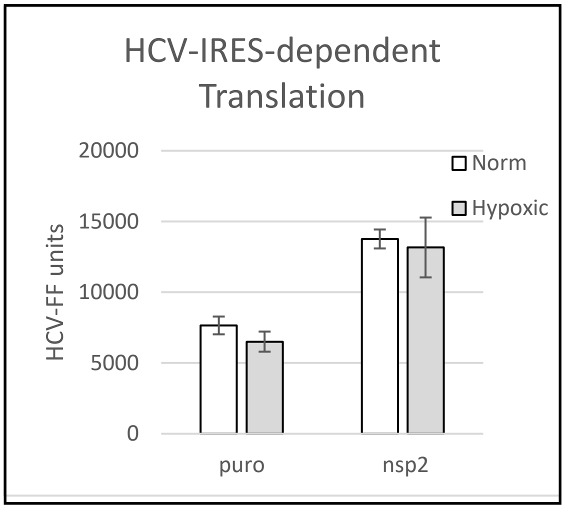

Supplemental Figure 3. Loading controls for the experiments described in Fig. 7B.

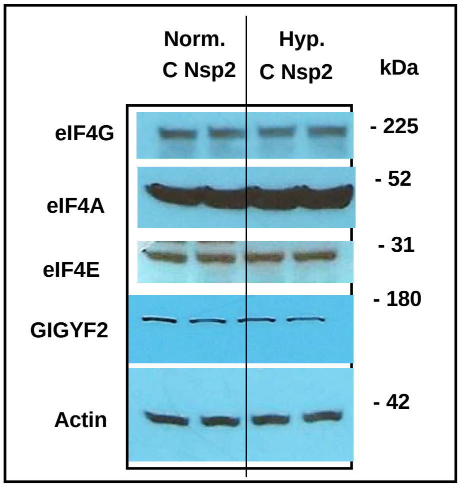

Supplement: Supplementary file 1 — Additional file 1: Figure S1. Measurement of Protein Synthesis rates in A549 cells depleted of eIF4E2. A549 cells have been transfected with siRNA (SENSE: CGAGACAAGAAUCAGAGCAtt, Ambion/Life Technologies) for 24 h. The inset shows the effective dose-depletion of eIF4E2. 105 A549 cells with or without 4E2-siRNA were each plated in 12 wells of a 24-well plate with 1 ml complete D-MEM and 10% FCS (duplicate samples). The next day the medium was replaced with medium containing 5 μCi/ml L-[3,4,5-3H(N)]-Leucine (150 Ci/mmol—NEN), and sequential aliquots were removed at indicated intervals. After solubilization with 0.5 ml 1%SDS, 10% TCA insoluble material (0.1 mg Protein) was collected on 2.5 cm GFA filter. Following washing with 90% EtOH, the filters were air-dried and placed in scintillation vials for counting with OptiScint LLT NPE-Free Scintillation cocktail in a Beckman LS6500 counter. Note that the difference in PS rates is highly significant (P < 10−6). 10 µg of protein isolated at 1 h was processed by 8%PAGE/SDS for fluorographic auradiography with PPO. Figure S2. Nsp2-cells demonstrate higher cap- and HCV-dependent translation under normal and hypoxic conditions. Expression of Renilla and Firefly luciferases from capped-Renilla-HCV-IRES-FF Luciferase mRNA in control and Nsp2 cells grown under normal (white bars) or hypoxic (grey bars) conditions. Graphs represents mean of Renilla units (expressed from the capped mRNA) (left panel), and Firefly (expressed from the HCV IRES mRNA) (right panel) luciferase signals (± SD, n = 3). Figure S3. Loading controls for the experiments described in Fig. 7B. [file 12985_2023_2021_MOESM1_ESM.pdf]
