# Supplementary material for: SARS-CoV-2 viral protein Nsp2 stimulates translation under normal and hypoxic conditions
Source: Virol J. 2023 Mar 30;20:55. doi: 10.1186/s12985-023-02021-2 (PMC10060939; doi:10.1186/s12985-023-02021-2)
Supplement: Supplementary file 2 — Additional file 2: Table S1. Primers used for real-time qPCR to analyze total RNAs and RNAs in polysomal gradients. Related to the STAR Methods. [file 12985_2023_2021_MOESM2_ESM.docx]

**SUPPLEMENTAL INFORMATION**

**Table S1. Primers used for real-time qPCR to analyze total RNAs and RNAs in polysomal gradients. Related to the STAR Methods.**

| **Target gene** | **sequence** | **source** |
| --- | --- | --- |
| GAPDH-fwd | GTC AGC CGC ATC TTC TTT TG | IDT, Morrisville, NC |
| GAPDH-rev | GCG CCC AAT ACG ACC AAA TC | IDT, Morrisville, NC |
| FGF2 | Mix of forward and reverse primers, Cat# HP100111 | Sino Biological US |
| FF-Luc-fwd | TAT CCG CTG GAA GAT GGA AC | IDT, Morrisville, NC |
| FF-Luc-rev | ATA AAT AAC GCG CCC AAC AC | IDT, Morrisville, NC |
| VEGF-C-fwd | TTT GCC AAT CAC ACT TCC | MWG-Biotech AG |
| VEGF-C-rev | TTG TTC GCT GCC TGA CAC | MWG-Biotech AG |
| 18S-fwd | CTA CCA CAT CCA AGG AAG CA | IDT, Morrisville, NC |
| 18S-rew | TTT TTC GTC ACT ACC TCC CCG | IDT, Morrisville, NC |
